# Does Early Childhood Vaccination Protect Against COVID-19?

**DOI:** 10.3389/fmolb.2020.00120

**Published:** 2020-06-05

**Authors:** Karzan R. Sidiq, Dana Khdr Sabir, Shakhawan M. Ali, Rimantas Kodzius

**Affiliations:** ^1^Charmo Centre for Research, Training and Consultancy, Charmo University, Chamchamal, Iraq; ^2^Department of Medical Laboratory Sciences, Charmo University, Chamchamal, Iraq; ^3^Department of Oral and Maxillofacial Surgery, University of Sulaimani, Sulaimani, Iraq; ^4^Panevezys Faculty of Technology and Business, Kaunas Technology University (KTU), Panevezys, Lithuania; ^5^Faculty of Medicine, Ludwig Maximilian University of Munich (LMU), Munich, Germany

**Keywords:** children, virus, COVID-19, measles, rubella, immunity, vaccination

## Abstract

The coronavirus disease 2019 (COVID-19) is an on-going pandemic caused by the SARS-coronavirus-2 (SARS-CoV-2) which targets the respiratory system of humans. The published data show that children, unlike adults, are less susceptible to contracting the disease. This article aims at understanding why children constitute a minor group among hospitalized COVID-19 patients. Here, we hypothesize that the measles, mumps, and rubella (MMR) vaccine could provide a broad neutralizing antibody against numbers of diseases, including COVID-19. Our hypothesis is based on the 30 amino acid sequence homology between the SARS-CoV-2 Spike (S) glycoprotein (PDB: 6VSB) of both the measles virus fusion (F1) glycoprotein (PDB: 5YXW_B) and the rubella virus envelope (E1) glycoprotein (PDB: 4ADG_A). Computational analysis of the homologous region detected the sequence as antigenic epitopes in both measles and rubella. Therefore, we believe that humoral immunity, created through the MMR vaccination, provides children with advantageous protection against COVID-19 as well, however, an experimental analysis is required.

## Introduction

The coronavirus disease 2019 (COVID-19) is a contagious viral infection of the respiratory system caused by SARS-coronavirus-2 (SARS-CoV-2). The outbreak of the disease was first reported in Wuhan, China in December 2019 (Ralph et al., 2020; Wu F. et al., 2020). The spread of COVID-19 is continuous and was declared a pandemic disease by the World Health Organization (WHO) on 11 March, 2020 (World Health Organization, 2020a). As of 20 May 2020, more than 4.7 million people have contracted the disease and 318,789 people have died (World Health Organization, 2020b).

**Figure 1:**
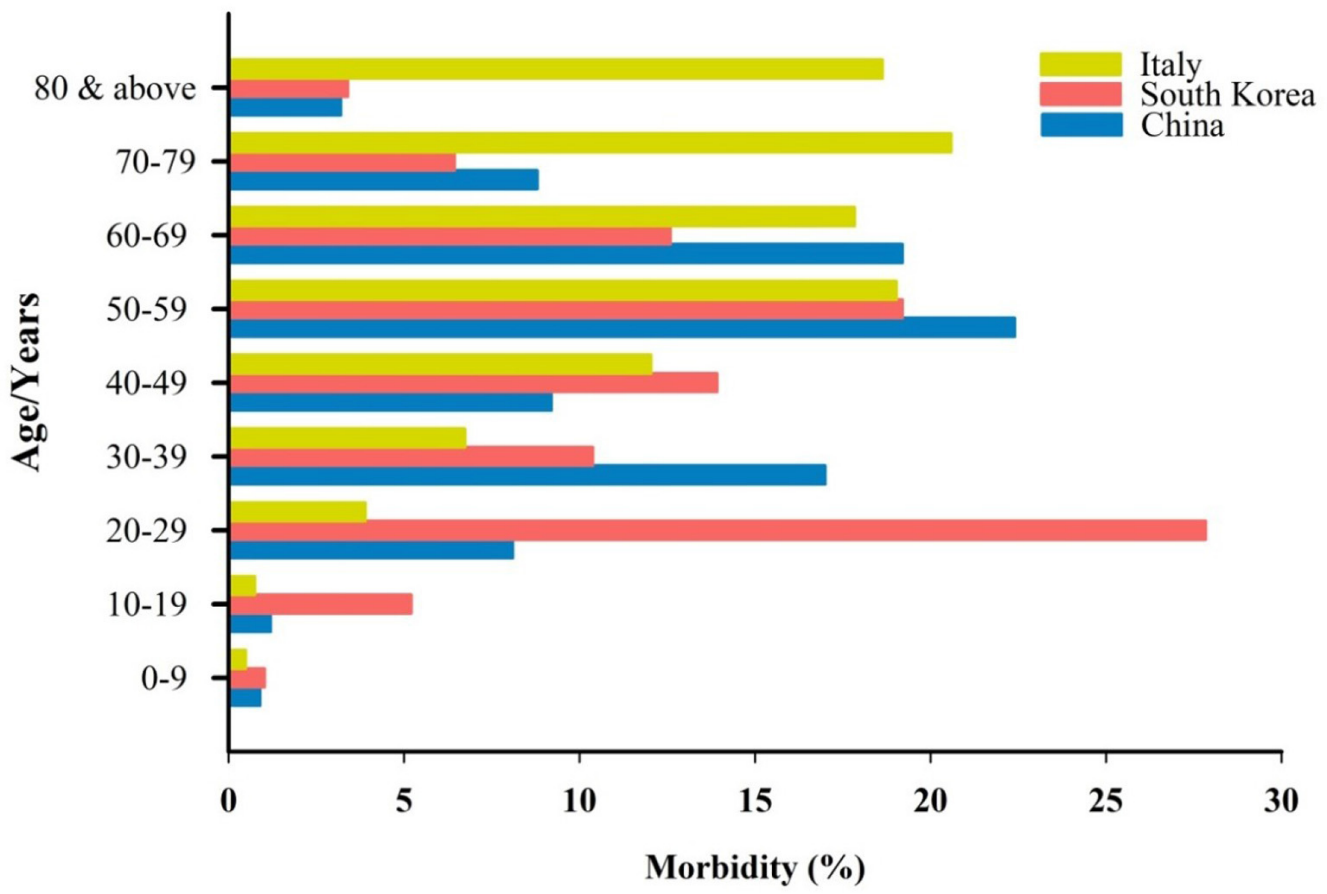
Age distribution and the percentage of morbidity by COVID-19 in three different countries.

Detailed data of COVID-19-infected patients from China, Italy, and South Korea have shown that the disease is less common and milder in children younger than 10 years of age (Figure 1) [Carrozzi et al., 2020; Epidemiology Working Group for NCIP Epidemic Response, 2020; Korean Centers for Disease Control and Prevention (KCDC), 2020; Xu et al., 2020]. In China, only 0.9% of 72,314 infected people were children with a 0% mortality [China, 2020; Epidemiology Working Group for NCIP Epidemic Response, 2020; Korean Centers for Disease Control and Prevention (KCDC), 2020]. Cascella et al. (2020) also pointed out that children are less affected by the disease, but they might be a carrier, transmitting the disease to other people. A recent study conducted among 2,143 confirmed and suspected cases in China. It was observed that infants (<1-year-old) are vulnerable to the disease, however, the symptoms of COVID-19 are generally milder to medium among children (Dong et al., 2020).

The Korean Centres for Disease Control & Prevention (KCDC) released the data of a total of 8,413 COVID-19 cases on 18 March 2020 [Korean Centers for Disease Control and Prevention (KCDC), 2020]. Similar to China, only 1.03% of the total of infected people were children aged below 10 years of age [Korean Centers for Disease Control and Prevention (KCDC), 2020]. Moreover, in Italy, 0.49% of the 24,879 people infected with COVID-19 were children younger than 10 years old (Carrozzi et al., 2020). The same pattern was seen in the data released about 2,449 known-age Covid-19 patients in the USA (CDC COVID-19 Response Team, 2020). There was not a single child recorded to be admitted to an intensive care unit (ICU) in the USA (CDC COVID-19 Response Team, 2020). Despite the low incidence of COVID-19 among children, it appears that the virus mostly affects adults and the elderly, particularly those who have other health problems [Epidemiology Working Group for NCIP Epidemic Response, 2020; Korean Centers for Disease Control and Prevention (KCDC), 2020; Remuzzi and Remuzz, 2020]. The reasons why children are less susceptible to COVID-19 remain unclear. Here, we hypothesized that the MMR vaccination might be a reason why children have protection against the disease.

## Coronavirus Particle

**Figure 2:**
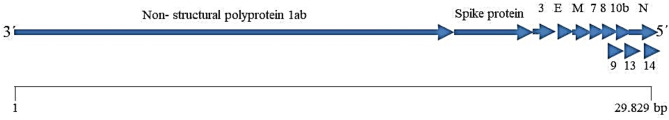
Schematic representation of the SARS-COV-2 genome (according Wu A. et al., 2020).

SARS-COV-2 is a single- strand, positive-sense RNA virus that is a member of the family Coronaviridae, alongside the severe acute respiratory syndrome (SARS) coronavirus (SARS-CoV) and the Middle East respiratory syndrome (MERS) coronavirus (MERS-CoV) (Lu et al., 2020). It has a genome size of nearly 29.9 kbp, encoded for nearly 12 ORFs including the Spike glycoprotein, envelope protein, and the membrane glycoprotein (Figure 2) (Wu A. et al., 2020). The Spike (S) glycoprotein is an important protein that plays a key role in the viral binding to an angiotensin-converting enzyme 2 (ACE2) receptor on the epithelial cells of the respiratory system (Wrapp et al., 2020). Therefore, the S protein is a key immunogenic protein of SARS-Cov-2 that induces the host immune system (Ahmed et al., 2020).

## Immunity and Immunization

The immune system fights off any foreign particles that enter the human body. The viral particles possess antigenic structures that respond through the adaptive immune system via antibody production. Antibodies are proteins that recognize the invading pathogens by binding specifically to their surface antigenic proteins. This antibody-antigen binding is important for neutralizing and the prevention of viral infections. So, viruses can be removed from the body by antibodies before they get the chance to infect a cell (Anaya et al., 2013; Thapa and Farber, 2019). The production of antibodies can actively be induced via immunization.

Humans are routinely immunized against several viral diseases like measles, rubella, mumps, hepatitis A, hepatitis B, rotavirus, and poliomyelitis in early childhood. These immunizations usually induce broad immunity against the viral particles, when the live-attenuated whole viral particles are used in the vaccines. We thought that the surface proteins of one or more of the above-mentioned viruses could share antigenic epitopes with the spike (S) glycoprotein of SARS-CoV-2. Therefore, the general immunity of children against COVID-19 could be related to the antibodies that are produced through routine vaccination. The antibodies may then cross-react with the antigenic epitopes of the spike (S) glycoprotein.

## Homology Sequence Searching for The SARS-CoV-2 Spike (S) Protein

**Figure 3:**
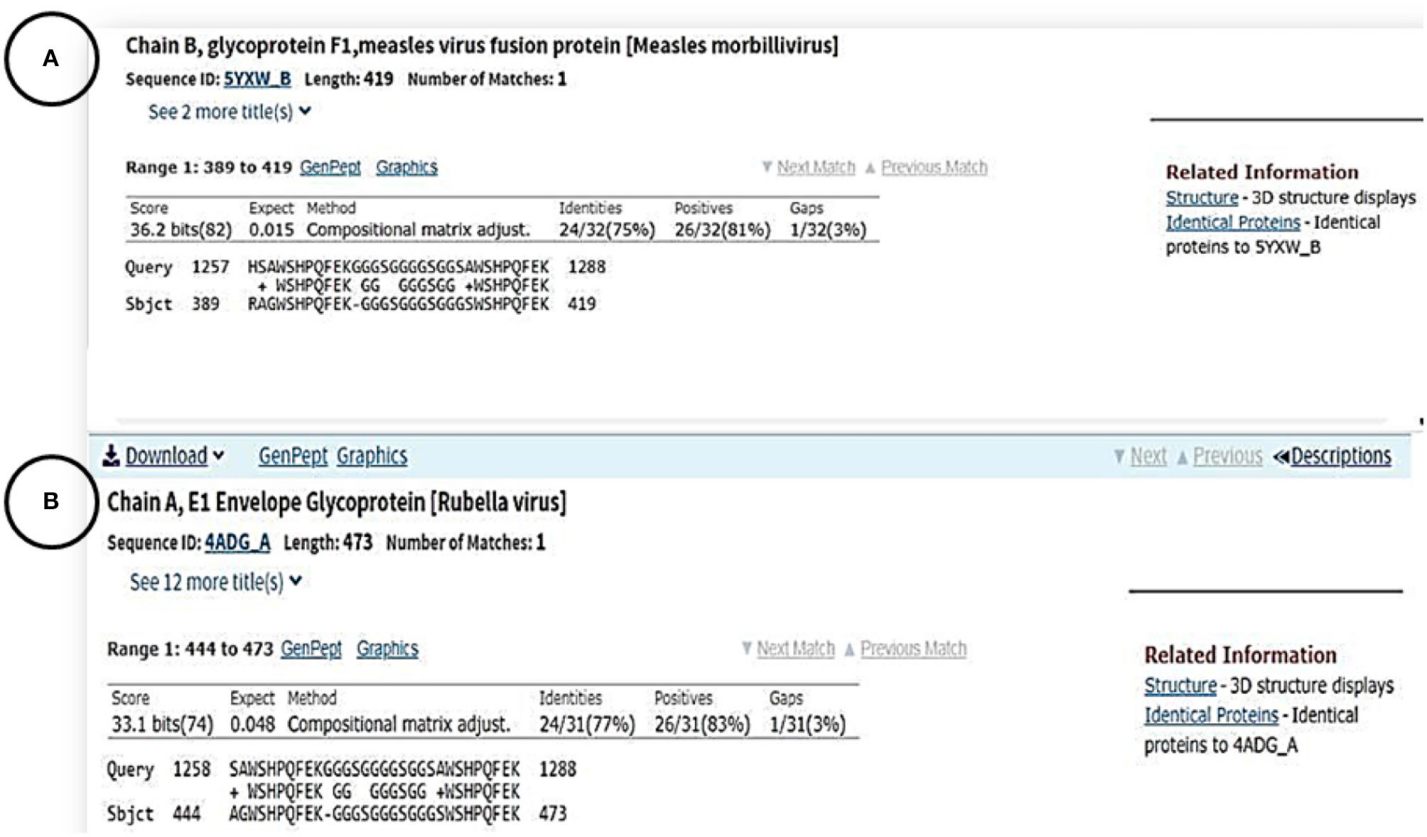
Amino acid sequence alignments between the surface protein of SARS-CoV-2 and chain B of glycoprotein F1 protein measles **(A)** chain A of E1 envelop glycoprotein in Rubella virus **(B)**.

To investigate whether or not routine child vaccinations provide protection against COVID-19, we performed a homology sequence search for the chain A amino acid sequence of the SARS-CoV-2 Spike (S) glycoprotein (PDB: 6VSB_A) against the proteomic sequences of vaccine-preventable viruses. Interestingly, we found that 30 amino acid residues share similarities between the Spike (S) glycoprotein of the SARS-CoV-2 virus and the Fusion (F1) glycoprotein of Measles virus (residues R389 to K419; Figure 3A) as well as with the envelope (E1) glycoprotein of the Rubella virus (residues A444 to K473; Figure 3B). Moreover, we used the computerized superimposed structure method to find any sequence similarities between the crystal structure of the SARS-COV-2 spike glycoprotein (Wrapp et al., 2020) and the crystal structure of the prefusion form of the measles virus fusion protein (PDB: 5YXW) (Hashiguchi et al., 2018). The result showed no similarity.

**Figure 4:**
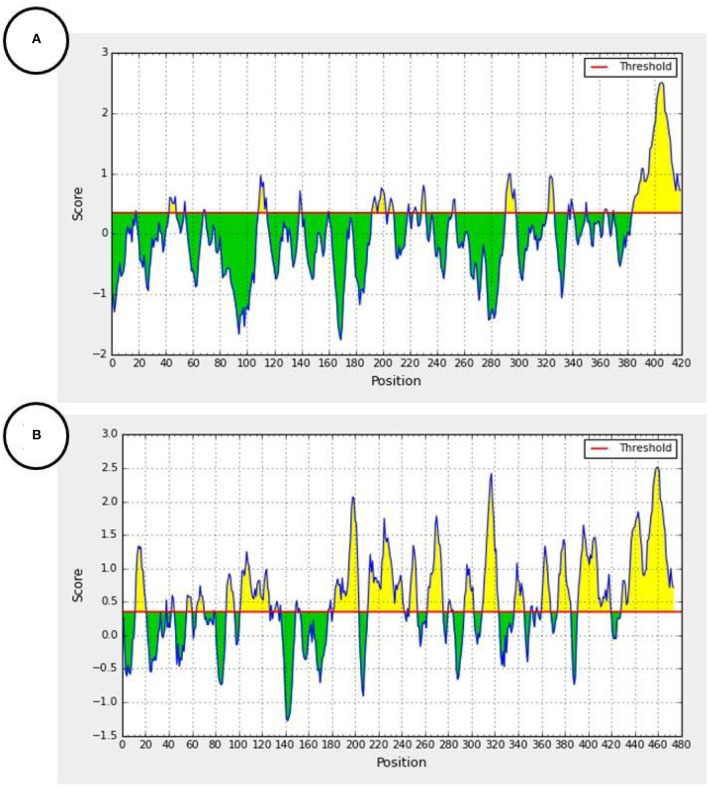
Predicted antibody epitopes of the F1 measles virus glycoprotein (Accession number: 5YXW_B) **(A)** and E1 Rubella Glycoprotein (Accession number: 4ADG_A) **(B)** using the iedb.org online tool (http://tools.iedb.org/bcell/). Yellow colored regions are predicted antibody epitopes of the protein.

However, this short homologous amino acid sequence appeared to have an epitope property, and is involved in antibody production, using the antibody epitope prediction online tool (http://tools.iedb.org/bcell/) (Figure 4) (Larsen et al., 2006; Ponomarenko and Bourne, 2007).

## Discussion

In terms of epidemiology, children and the elderly are two groups of people who are at high risk of contracting infectious illnesses. However, data released from COVID-19 patients show that children are less susceptible to contracting the disease, compared to adults and the elderly (Brodin, 2020; Cascella et al., 2020; Sabir et al., 2020). Understanding the immunological base of children's protection against COVID-19 can prevent and control further spreading the disease.

The shared immunity between vaccine-preventable childhood diseases and COVID-19 was based on the homology sequence similarity of the 30 amino acid residues between the Spike (S) glycoprotein of the SARS-CoV-2 virus with the Fusion (F1) glycoprotein of Measles virus (residues R3389 to K419), and the envelope (E1) glycoprotein of the Rubella virus (residues A444 to K473). The Spike glycoprotein protein of SARS-CoV-2, similar to SARS-CoV, has the same human cell receptor—the angiotensin-converting enzyme 2 (ACE2) (Hoffmann et al., 2020). The essential amino acids of the receptor binding motif of the SARS-CoV-2 S protein are the residues located between R319 – K529 (Wan et al., 2020). These residues make direct contact with the human cell receptor (ACE2) and then help to fuse the virus to the host cell membranes. However, the short 30 amino acids of the S SARS-COV-2 protein that show similarity to the measles protein were not located in this region; rather, they were at the end of the N-terminal of the S SARS-CoV-2 protein. We also did not find any other sequence similarity between the crystal structure of the SARS-COV-2 protein and the crystal structure of the prefusion form of the measles virus fusion protein (PDB: 5YXW]. Considering the location of these 30 residues, it is possible that these amino acids are exposed to the outside of the protein and have access to soluble proteins. Previously, a protein structure analysis of several different proteins concluded that the terminal residues of the proteins are generally exposed to the outside (Jacob and Unger, 2007).

In addition, according to the antibody epitope prediction online tool, the homology region in either the F1 Measles glycoprotein (Figure 4A) or the E1 Rubella virus envelope Glycoprotein (Figure 4B) can target antibodies and show antigenic epitopes hotspots of the proteins. Moreover, children are usually immunized against live- attenuated vaccines of measles and rubella. The production of a broad humoral immunity (polyclonal antibodies) against these viruses is feasible, when the whole viral particle is introduced to the immune system (Atabani et al., 1997). It was reported that the Fusion (F1) glycoprotein of the measles virus induces the production of neutralizing antibodies (Malvoisin and Wild, 1990; Atabani et al., 1997). Like the spike (S) glycoprotein of SARS-CoV-2, the envelope (E1) glycoprotein has a receptor-binding function, so it is the main antigen and sole target of neutralizing antibodies against the rubella virus (DuBois et al., 2013).

Immunization against measles and rubella in Italy, South Korea, and China date back to the last century (Choe and Bae, 2012; Bechini et al., 2013; Zheng et al., 2018). Previous studies have shown that the antibody titers against measles and rubella are high during childhood, whereas the titers decrease with age (Dai et al., 1991; Kontio et al., 2012). Each antigenic epitope constitutes 15 amino acid residues and only five of them strongly bind to the corresponding amino acids on the antibody paratopes (Frank, 2002). The chance of locating epitopes in the 30 amino acid residues that are shared between the COVID-19 virus, measles and rubella viruses is unknown. Moreover, like COVID-19, measles causes respiratory complications and is transmitted through sneezing and coughing droplets. The humoral immunity sharing was already reported between Zika and Dengue fever viruses (Montecillo-Aguado et al., 2019). Previously, it was also proposed that the winter flu among children could increase the level of antibodies that fight against other diseases including Covid-19 (Dong et al., 2020). However, not all children catch the winter flu and the flu vaccine is not globally applied to every child. Thus, our data suggest that the humoral immunity, produced through immunization against measles and rubella, may make children less susceptible to COVID-19 as well. However, experimental data are required to prove our hypothesis.

In summary, data published among the people infected people with COVID in China, Italy, and South Korea showed that children are generally less affected by the disease. We hypothesized that such immunity among children is related to early childhood vaccination, particularly anti-measles and anti- rubella vaccinations.

## Data Availability Statement

The original contributions presented in the study are included in the article/supplementary files, further inquiries can be directed to the corresponding author.

## Author Contributions

Manuscript was prepared by KS, DS, SA, and RK. Data analysis was performed by DS and RK. Final revision and approval was done by RK.

## Conflict of Interest

The authors declare that the research was conducted in the absence of any commercial or financial relationships that could be construed as a potential conflict of interest.
